# Erlotinib as a salvage treatment after gefitinib failure for advanced non-small-cell lung cancer patients with brain metastasis: A successful case report and review

**DOI:** 10.1097/MD.0000000000026450

**Published:** 2021-06-25

**Authors:** Yong Dong, Qijun Li, Qian Miao, Da Li

**Affiliations:** a Department of Medical Oncology, Sir Run Run Shaw Hospital, College of Medicine, Zhejiang University, Hangzhou; b Department of Medical Oncology, Quzhou People's Hospital, Zhongloudi, Quzhou, Zhejiang, China.

**Keywords:** case report, erlotinib, gefitinib, non-small-cell lung cancer (NSCLC), salvage treatment

## Abstract

**Rationale:**

The guidelines recommended gefitinib as a first-line targeted treatment for stage IV non-small-cell lung cancer (NSCLC) patients with EGFR mutations. However, resistance to gefitinib ensues invariably and there is little evidence as for the effectiveness of subsequent salvage treatment for patients without T790m mutation. The case is to evaluate the efficacy of erlotinib, another EGFR-TKI, after failed first-line use of gefitinib.

**Patient concerns:**

We described a 55-year-old man with good performance status (PS).

**Diagnoses:**

He was histopathologically diagnosed stage IV lung adenocarcinoma with EGFR mutations in November 2018.

**Interventions:**

He was administrated with gefitinib daily (250 mg) for activating epidermal growth factor receptor (EGFR) mutations (exon 19 deletions,19del), and combined with platinum-based dual-drug chemotherapy. During the target treatments, the optimal efficacy evaluation was partial remission (PR) with a 12-month progression-free survival (PFS) time. Later, the intracranial progression of the patient rendered the treatment change to erlotinib.

**Outcomes:**

It is surprising that the tumor lesion in brain as well as lung relieved obviously. His progression-free survival (PFS)was nearly 11 months, and the overall survival (OS)was>36 months up to now. The adverse events were tolerable.

**Lessions:**

This case manifests that re-biopsy of advanced or recurrent NSCLC is beneficial to make a better therapeutic regimen, and erlotinib can be used as a salvage treatment after gefitinib failure.

## 1 Introduction

Lung cancer is the most common cause of tumor associated death mortalities with the highest incidence.^[1]^ Generally speaking, the development of drugs and mechanism researches of drug resistances were always discovered firstly in lung cancer, and then gradually expanding application to other cancers. Although immunotherapy has developed rapidly and become an efficient treatment for many cancers, such as non-small cell lung cancer (NSCLC), malignant melanoma and other cancers,^[2,3]^ as treatments with more mature researches, chemotherapy and targeted treatments still play important roles in the oncotherapy. Epidermal growth factor receptor-tyrosine kinase inhibitor (EGFR-TKI) is a significant milestone in drug therapy, which is widely used in patients of NSCLC with constantly more accurate indications. Nowadays, EGFR-TKI has been administrated as a first-line treatment for NSCLC in stage IV with EGFR mutations, and it demonstrates a better efficacy compared to traditional chemotherapy in the selected patients.^[4,5]^ Drugs are rapidly updated, in terms of EGFR-TKI, includes the first generation of gefitinib, erlotinib and icotinib, the second generation of afatinib, dacomitinib and the third generation of osimertinib. However, most tumors inevitably develop acquired resistance to EGFR TKIs over time, which limits the benefits of patients and makes patients eventually undergo disease progression.^[6,7]^ How to make rational administration of drug therapy, such as the combination of drugs to reduce the occurrence of drug resistance, the indication of continuing the original TKI or dressing change treatments after drug resistance, is our grand challenges in tumor therapy. The correct strategy of EGFR-TKI treatments can maximize the survival time of patients, while the implicit mechanism of EGFR-TKI resistance is the major obstacle which need a plenty of case reports and clinical trials to illustrate and point out the research direction. This paper reports a patient of primary lung adenocarcinoma with brain metastasis who developed drug resistance after first-line gefitinib combined with chemotherapy, and erlotinib succeeded in complete remission of intracranial lesions and partial remission of pulmonary lesions as the second-line treatment. In the meanwhile, we expounded the phenomenon of no cross-drug resistance among the first generation of EGFR-TKI and related clinical problems in the treatments of lung cancer complicating with brain metastasis.

## 2 Case presentation

**Figure 1:**
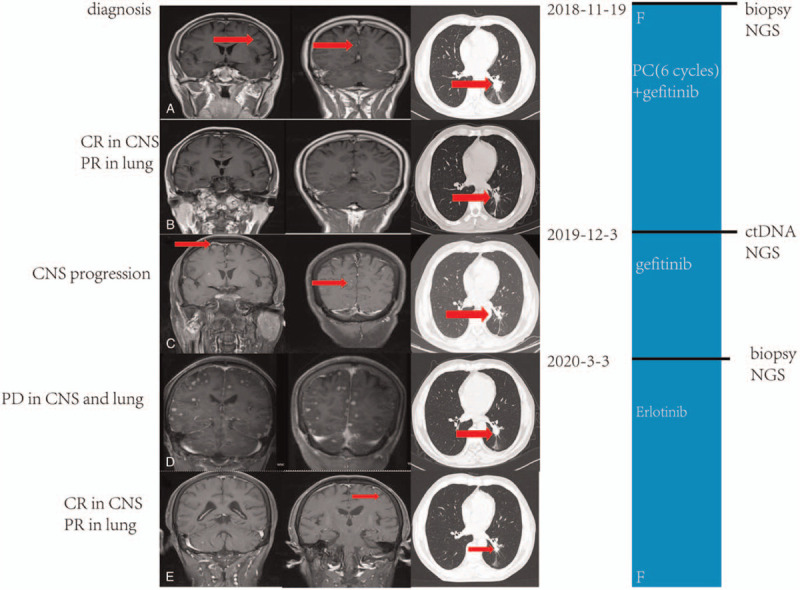
Timeline and effect of EGFR-TKI treatments. (A) Baseline images of CT and MRI at diagnosis; (B) Brain metastases complete remission (CR) and original lung mass partial response (PR) after six cycles of PC (pemetrexed 1 g and carboplatin 500 mg) and one year of gefitinib treatment; (C) Brain metastases progressed gefitinib treatment was administered; (D) Images of CT and MRI before erlotinib treatment; (E) Brain metastases CR and lung mass PR after erlotinib treatment was administered; (F) Treatment timeline; CNS = central nervous system; CT = computed tomography; EGFR, epithelial growth factor receptor; MRI = magnetic resonance imaging; PD = progressive disease; TKI = tyrosine kinase inhibitor.

A 55-year-old man without the history of smoking or related pulmonary diseases previously was presented to hospital for coughing with sputum, chest distress and breathless in November 2018. Computed tomography (CT) scans showed a lung mass of the lower left lobe, and brain magnetic resonance imaging (MRI) revealed brain metastases in left frontal lobe and insula (Figure 1). Percutaneous lung biopsy confirmed adenocarcinoma on November 19, 2018, and he was clinically diagnosed with stage IVa lung adenocarcinoma (cT1cN1M1b) with an EGFR 19del mutation, detected through the Next Generation Sequencing (NGS) platform. Variant allele frequencies (VAFs) of the detected EGFR 19del mutation were 19.6%. Gefitinib (250 mg, once a day) was then administered from December 3, 2018. After one month of gefitinib treatment, chest CT scans on December 31^st^ 2018 showed original lung mass was significantly reduced, and the brain metastases almost disappeared, which were evaluated partial remission (PR) and complete remission (CR) respectively according to the Response Evaluation Criteria in Solid Tumors (RECISTv1.1) (Figure 1). During the treatment of gefitinib, the patients received PC (pemetrexed 1 g and carboplatin 500 mg) regimen for 6 cycles (from 2019-01 to 2019-06), and maintain with single pemetrexed (1 g) chemotherapy for 5 cycles (from 2019-07 to 2019-11) in Shanghai Thoracic Hospital. Regular follow-up shows the therapeutic effects PR. Unfortunately, in December 3, 2016, a new craniocerebral metastasis was discovered by brain MRI. According to the CSCO guidelines, oligoprogression or only progression in central nervous system (CNS) of NSCLC with EGFR mutation in stage IV after drug resistance, the original EGFR-TKI and local treatment can be continued, and the mechanism of drug resistance can be confirmed by biopsy again. Therefore, the patient explored the genetic mutation through hematological tests for his unpuncturable lung lesion. He finally was administered with gefitinib continually with no EGFR mutation detected, combined with brain intensity modulated radiation therapy (IMRT) (DT 3000cGy/10F whole brain) from December 18, 2019 to December 31, 2019. The patient sustained the treatment of gefitinib until March 3, 2020, when brain MRI indicated that multiple metastases in brain were in explosive development, and chest computed tomography (CT) showed masses in lung were significantly larger than the baseline. The overall evaluation of the efficacy was progressive disease (PD), and it is recommended to change treatments after biopsy according to the guidelines. In our perspective, it takes time for obtaining the result of re-biopsy and NGS, and the huge tumor load and rapid progress in intracranial tumor made the waiting long and tough. Ultimately, targeted therapy of erlotinib (150 mg once a day) was administered from March 20, 2020, and no bevacizumab combined with temporarily for biopsy preparation. On March 24, 2020, percutaneous lung biopsy under CT was performed. Pathology revealed lung adenocarcinoma and NGS identified the molecular profiling of c.2240_2254delp.L747_T751del (13.8%), TP53 (28.98%), MSS, PD-L1 (-) without T790 M mutation. In the meanwhile, it was miraculously found that brain MRI on April 22, 2020 indicated a PR status in brain metastases. Therefore, the patient continued administering with erlotinib and was performed regular follow-ups thereafter. Up to March 31^st^, 2020, the intracranial and pulmonary metastases were almost evaluated as CR.

## 3 Discussion

The emergence of small molecular targeted drugs revolutionized the therapeutic strategies of NSCLC with positive driving genes. Although the first-generation EGFR TKIs is more effective than conventional chemotherapy in patients with EGFR mutations, the median progression-free survival (PFS) of most patients is about 1 year due to ubiquitously acquired drug resistance in targeted drugs.^[8]^ At present, the most common acquired resistance mechanisms of EGFR TKI have been explored out includes alternative pathway activation, such as epithelial mesenchymal transformation (EMT), mesenchymal epithelial transformation (MET) or ERBB2/HER2 amplification, tumor histological as well as phenotypic transformation, and especially target gene modification like T790 M.^[9–11]^ With the development of NGS, more and more mutations related with drug resistance to EGFR TKIs have been revealed. In addition to the T790 M mutation, which accounts for about 50% of the drug resistance mechanism,^[12]^ other rare drug-resistant mutations includes insertion mutations in the site of Pro772_His773insGlnCysPro in exon 20, which can lead to resistant to erlotinib,^[13]^ mutations of codon 719 and 709 in exon 18 often indicate resistance or low response to first-generation EGFR TKIs.^[14]^ Compared with other rare mutations, patients with T790 M mutation tend to have a longer survival time for it is the only drug-resistant mutation which we have found a targeted drug.^[15]^ While the subsequent therapy after the first-generation EGFR TKIs resistant for patients without T790 M mutation is still a problem difficult to overcome. In most instances, doctors will advise patients receive systematic chemotherapy, and there are also plenty of clinical studies on the combination of chemotherapy and immune checkpoint inhibitors (ICIs), or anti-vascular endothelial growth factor receptor (VEGFR) carried out. In fact, before the third-generation EGFR TKIs developed, a certain amount of small samples clinical studies abroad confirmed that after the failure of advanced NSCLC with gefitinib, erlotinib can achieve a good effect with 5–10% PR and 40–60% SD.^[16,17]^ Why there are different efficacy and cross-resistance spectrum among first-generation EGFR TKIs? What is the drug resistance mechanism? How to select the beneficiaries? On account of that NGS was not prevalent at that time, definite answers still remain to be solved.

According to the characteristics of disease progression, it can be divided into three subtypes, including oligoprogression, systemic progression and central nervous system (CNS) sanctuary progression,^[18]^ and based on the duration of disease control as well as clinical symptom, the diversity of EGFR-TKI failure could be categorized into three modes, including dramatic progression, gradual progression and local progression.^[19]^ In our case, there were two disease progression during the course of the treatments.

1. The patient was administered with gefitinib combined with chemotherapy as the first-line treatments, whose PFS reached 12 months, which was significantly shorter than the combined group of prospective randomized controlled trials NEJ009, whose median PFS reached 20.9 month.^[20]^ The baseline status complicated with brain metastasis may account for the reduced PFS, and the developed brain metastasis firstly came after gefitinib resistance confirmed our consideration. The research results of IMPRESS showed that there was no clinical benefit from the continuation of EGFR TKIs after the first-line EGFR TKIs resistant in advanced NSCLC patients with EGFR mutations, while in the subtypes with negative T790 M mutation, there was a tendency to benefit.^[21]^ Because the residual lesion in the lung was too small to be punctured, we performed a liquid biopsy and the result showed no T790 M mutation. Combined with the slow and local CNS progression of the patient, we ultimately chose to continue the original TKI treatment plus craniocerebral local radiotherapy to control the disease progression.
2. The second progression of the patient was extensive and explosive progression in both brain and lung. Although we considered giving the patient a re-biopsy of the primary lung lesion to acquire a more precise treatment, the disease progressed fiercely, and the patient was administered with erlotinib while waiting for the results of genetic testing.

Fortunately, the patient had a good response under our treatment strategy.

During the course of treatments, we performed totally three times of genetic testing:

1. The genetic test of the punctured lung tissue in the initial state of disease indicated EGFR19 del, and we chose gefitinib for no osimertinib available in domestic market at that time, and the OS of the FlURA clinic trial hadn’t been published.
2. We performed a fluid biopsy immediately after the disease progressed under the treatment of gefitinib. Fluid biopsy could avoid repeated biopsies and monitor the changes of clones in real time, which detected drug resistance mutations of EGFR TKIs early and reduce missed mutations due to tumor heterogeneity.^[22]^ However, plasma genotyping had a false negative rate of 30 percent,^[23]^ and ignored the resistance mediated by histological transformation.^[24]^ Therefore, patients whose fluid biopsies indicate negative drug resistance mutations still need further tumor biopsies to be combined with, which assisted in confirming the mechanism of drug resistance. However, in our case, it is worth noting that patient's local progression first to appear in CNS, and fluid biopsy became the only way to determine the cause of drug resistance for the limited biopsy technique of CNS in this case.^[25]^ Due to the existence of blood-brain barrier, secondary systemic drug resistance mutations were not the only explanation for CNS progression. Given that the difficulty of drug penetration into the CNS, tumor tissue may still remain sensitive to EGFR TKIs.^[26,27]^ No EGFR mutation was detected in the patient, and we consider that it may be a false negative situation for concentration of ctDNA in peripheral blood too low to be detected after treatments, who had to rely on tissue biopsy for confirming the resistance mechanism.
3. After the progression of the lung lesion, we had the opportunity to biopsy again. NGS of tumor tissues showed EGFR mutation again, without drug resistance mutation, and combined with a new mutation of TP53.

**Figure 2:**
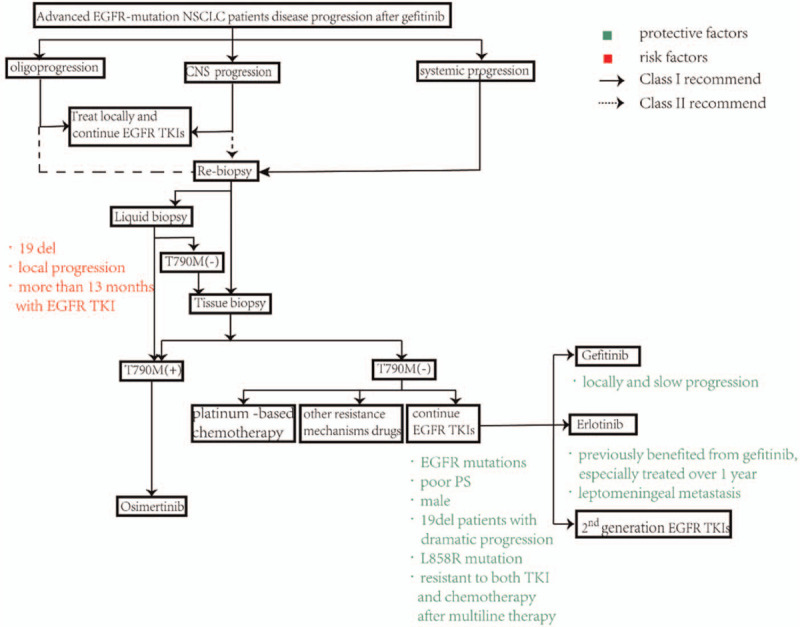
Treatment algorism and influence factor for advanced EGFR-mutant NSCLC patients disease progression after gefitinib. CNS = central nervous system; PS = performance status.

Up till now, there is no targeted drug aiming at TP53 mutation, and it is more commonly served as a predictive marker of best beneficiary population. Patients with EGFR mutations and wild-type TP53 benefit more from the combination treatments of EGFR-TKIs and bevacizumab,^[28]^ which may be related to the fact that TP53 mutations are a poor prognostic factor in patients with TKI and participate in primary drug resistance of EGFR-TKIs by small-cell lung cancer (SCLC) transformation.^[29–31]^ The detection of NGS during the treatments showed continuous change of EGFR mutation, which may be related to the dynamic regulation of drug resistance phenotype. Some studies have shown that the emergence of drug resistance may be short and reversible, and EGFR-TKI holiday can make lung cancer cell lines regain the sensitivity to EGFR TKIs.^[32–34]^ However, stopping EGFR TKI treatment prematurely may lead to tumor regeneration or rapid progression.^[35,36]^ Therefore, it is often believed that when patients are assessed as oligoprogression according to RECIST, the best option is to continue the original TKI, delay the time of changing drugs, and overcome the small part of drug resistant subclones by combining with local therapy.^[37–39]^ As the first-generation of TKIs, both gefitinib and erlotinib had the same action mechanism through combining ATP binding site reversibly,^[40]^ but erlotinib had more survival benefits than gefitinib as first-line treatment.^[41]^ In terms of pharmacology, the standard dose of erlotinib is the maximum tolerated dose (MTD), while the standard dose of gefitinib is about one third of its MTD,^[42]^ let alone erlotinib has higher steady state concentration and bioavailability.^[43]^ Therefore, there should be clinical benefits from erlotinib after gefitinib resistance. However, some studies on erlotinib after gefitinib failure showed that the overall effective rate was less than 10%. Further exploration by stratifying indicated that groups previously benefited from gefitinib were more likely to benefit from erlotinib, especially in patients underwent gefitinib more than one year,^[44]^ which may be caused by the bias of better disease status and tumor stability in patients who can tolerate long-term treatment of gefitinib.^[44]^ In the meanwhile, the cross resistance with gefitinib caused by acquired EGFR T790 M mutation or bypass oncogene MET amplification may also account for the failure of erlotinib after gefitinib resistance,^[45]^ especially in patients receiving EGFR TKIs lasting for more than 13 months, which is the independent factor of secondary T790 M mutation.^[46]^ Therefore, the clinical benefit can be obtained by continuing the treatment of erlotinib after gefitinib failure without cross resistance. In patients without T790 M mutation after first-line TKIs failure, OS of chemotherapy group was better than gefitinib combined with chemotherapy group. However, this study only demonstrated the comparation with original TKI, some studies suggest that switched gefitinib to erlotinib after gefitinib failure can get benefit. In this case, the patient had multiple brain metastasis as well as systemic progression, and the abundance of EGFR mutation was less than that before. However, the patient still got better clinical benefits under the treatment of erlotinib. Therefore, after the first-line of gefitinib resistance, in the case of oligoprogression in CNS without drug resistance mutations, it a better treatment to switch to erlotinib as soon as possible, for erlotinib's higher biological concentration and better permeability of the blood-brain barrier. And for NSCLC patients with leptomeningeal metastasis, it has been confirmed that erlotinib may improve their clinical outcome after initial failure of gefitinib and apatinib.^[47]^ We summarized the treatment process after gefitinib resistance and the characteristics of patients suitable for continuing EGFR-TKI treatment (Figure 2). Previous studies have shown that patients who has better performance status (PS) scores, a long-term response of (longer PFS) or washout period of chemotherapy between gefitinib and erlotinib, can benefit from erlotinib after gefitinib failure.^[48–50]^ Nowadays, with the widespread development of NGS, second biopsies to identify genetic status after gefitinib treatment make the beneficiaries of second-line erlotinib treatment found with more possibilities. We hanker for prospective randomized controlled trials to look for more evidence to support our inference.

## 4 Conclusion

Generally speaking, our case report suggested that when CNS progresses without drug resistance mutation indicated by NGS during the first-line use of gefitinib, switching to erlotinib as soon as possible may be a well treatment. In addition, it was also confirmed that repeated evaluation of drug-resistant mutations in the course of TKI treatment is indispensable, which provides ideas for stratifying the best beneficiary population of the TKI re-challenge.

## Author contributions

**Conceptualization:** Da Li.

**Data curation:** Yong Dong.

**Formal analysis:** Yong Dong.

**Investigation:** Yong Dong.

**Resources:** Qijun Li.

**Supervision:** Qian Miao.

**Writing – original draft:** Yong Dong, Qian Miao, Qijun Li.

**Writing – review & editing:** Da Li.
